# DUTCH weight control in atrial fibrillation study (DUTCH-WAIST): Design and rationale of a randomised controlled trial

**DOI:** 10.1007/s12471-026-02074-2

**Published:** 2026-09-14

**Authors:** Leonard Voorhout, Ron Pisters, Menno Huisman, Erik Klok, Frans Rutten, Geert-Jan Geersing, Robert Tieleman, Jan Hein Cornel, Astrid Schut, Jan Tijssen, Arianne van Bon, Martin Hemels

**Affiliations:** 1 Department of Cardiology, Rijnstate, Arnhem, The Netherlands; 2 Department of Medicine—Thrombosis and Hemostasis, Leiden University Medical Center, Leiden, The Netherlands; 3 Department of General Practice & Nursing Science, Julius Center for Health Sciences and Primary Care, University Medical Center Utrecht, Utrecht University, Utrecht, The Netherlands; 4 Department of Cardiology, Martini Hospital, Groningen, The Netherlands; 5 Department of Cardiology, Radboud University Medical Center, Nijmegen, The Netherlands; 6 Department of Cardiology, Northwest Clinics, Alkmaar, The Netherlands; 7 Dutch Network for Cardiovascular Research (WCN), Utrecht, The Netherlands; 8 Department of Internal Medicine, Rijnstate, Arnhem, The Netherlands

**Keywords:** Atrial fibrillation, Weight loss, GLP‑1 agonist, Rhythm control, Randomised controlled trial

## Abstract

**Background:**

Obesity is a well-established and highly prevalent risk factor for atrial fibrillation (AF) and its progression by promoting structural and electrical atrial remodelling, leading to AF recurrence and persistence. Weight loss is expected to reverse atrial remodelling and improve rhythm control. Optimal strategies for weight management are, however, under-explored.

**Objective:**

To quantify the effect of clinically meaningful weight loss on rhythm control in overweight patients with first detected persistent AF.

**Design:**

A multicentre, double-blind, randomised, placebo-controlled trial evaluating semaglutide 2.4 mg once weekly versus placebo, in addition to combined lifestyle intervention (CLI), during a one-year period.

**Participants:**

Adults with a BMI > 30 kg/m^2^, or BMI > 27 kg/m^2^ with a weight-related comorbidity, and first detected (< 6 months) persistent AF scheduled for electrical cardioversion.

**Intervention:**

Weekly subcutaneous injections of semaglutide 2.4 mg or placebo, alongside CLI and standard AF care in both groups.

**Outcomes:**

The co-primary outcome is (i) a hierarchical assessment of rhythm status at one year, ranking patients by severity from arrhythmic death to sinus rhythm without any additional rhythm control support. (ii) the composite of either persistent atrial fibrillation on ECG, catheter ablation for AF, arrhythmic death, or any serious adverse event due to anti-arrhythmic drugs occurring within 1 year.

**Conclusion:**

This study investigates whether treatment of overweight with semaglutide, as an adjunct to lifestyle intervention, enhances rhythm control in AF patients with first detected persistent AF and overweight, offering a potential new approach to reducing AF burden and cardiovascular risk in this population.

**Supplementary Information:**

The online version of this article (https://doi.org/10.1007/s12471-026-02074-2) contains supplementary material, which is available to authorized users.

## Introduction

Atrial fibrillation (AF) is the most common sustained arrhythmia in the Western world, ultimately affecting one in five adults [1]. The obesity epidemic importantly contributes to the lifetime AF risk [2], increasing its occurrence to more than one in three [1], with each BMI unit above normal weight raising the relative risk of developing AF by around 4% [3]. AF increases the global health burden through a significant risk of stroke, heart failure (HF), and all-cause mortality [4]. AF-related symptoms, including palpitations and dyspnoea, are common and can reduce quality of life and drive a rhythm control strategy, including invasive procedures such as ablation [4]. At the time of diagnosis, AF is frequently persistent rather than self-terminating [5], and if symptomatic, attempting sinus rhythm restoration via electrical cardioversion (ECV) is the current cornerstone of a rhythm control strategy [4]. However, maintaining sinus rhythm remains challenging: recurrent persistent AF following ECV occurs in nearly half of patients within one month and 60% after one year [6, 7]. Supportive rhythm control using anti-arrhythmic drugs (AADs) and ablation aims to prolong AF-free survival, but risks life-threatening side effects and procedural complications [8, 9]. Moreover, its long-term success remains limited if the underlying cause is unaddressed [10–12].

Obesity is believed to promote AF via cardiometabolic abnormalities, including chronic inflammation [13] and atrial remodelling [13, 14], predominantly expressed by left atrial (LA) dilatation [3]. LA enlargement is an important predictor of recurrence of persistent AF after an initial successful cardioversion [12]. Observational evidence demonstrated that weight reduction (≥ 10%) induces a reduction of AF symptom burden, [15] prolongs freedom from AF without the need for AADs and/or ablation [16], and is beneficial in atrial remodelling [15, 16]. These observations support the hypothesis that atrial fat begets AF [17], and weight reduction could be an innovative, potent rhythm control strategy as it targets the root cause of the arrhythmia, as opposed to AAD which focuses on secondary cardiac effects. Yet, the clinical effectiveness of weight reduction on restoring sinus rhythm is understudied in the management of (persistent) AF.

Until recently, only combined lifestyle interventions (CLI) were available for weight reduction. Although they remain an obvious starting point of any weight control attempt, solely CLI appears to be unable to achieve clinically meaningful, i.e., ≥ 5–10%, and persistent weight-loss in the majority of patients [18, 19]. However, the use of incretin-based therapies such as glucagon-like peptide (GLP)-1-agonists in adults with obesity opens a very interesting new avenue [21]. Importantly, studies have not explicitly included patients with persistent AF, making the cardiovascular impact of GLP‑1 agonists in AF patients unknown [22, 23]. We therefore designed a double-blind, randomised, placebo-controlled trial to investigate whether semaglutide 2.4 mg administered once weekly, in addition to standard care including CLI, improves sinus rhythm maintenance in overweight and obese adults with persistent AF.

## Methods

### Design

**Fig. 1:**
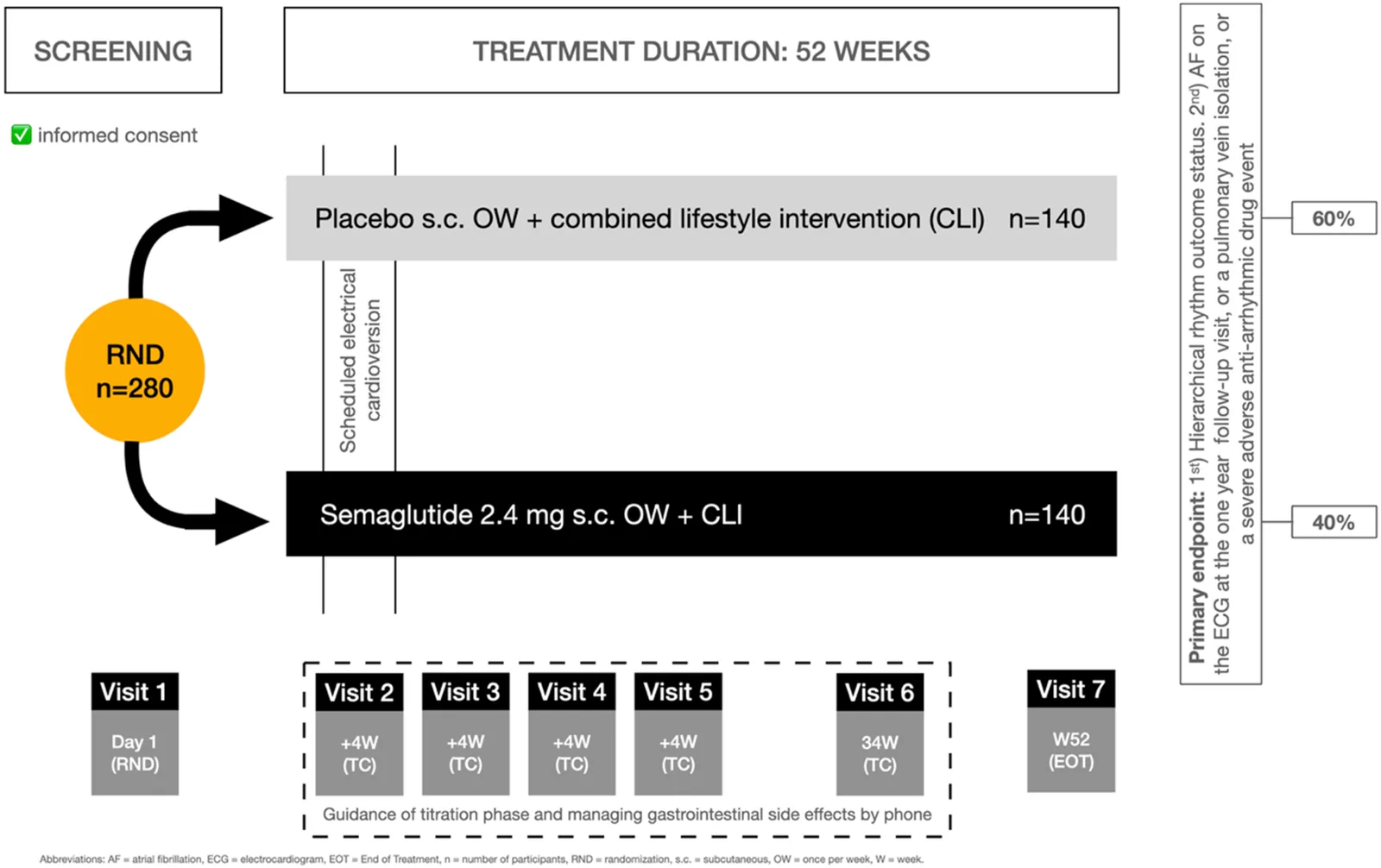
Flowchart. *AF* atrial fibrillation, *ECG* electrocardiogram, *EOT* End of Treatment, *n* number of participants, *RND* randomisation, *s.c.* subcutaneous, *OW* once per week, *W* week, *TC* telephone consultation

DUTCH-WAIST is an investigator-initiated, multicentre, double-blind, placebo-controlled, randomised trial with parallel groups, conducted across 21 hospitals in the Netherlands, including 18 WCN-affiliated non-academic hospitals and three academic medical centres. Randomisation will occur in a 1:1 ratio between two arms: once-weekly subcutaneous semaglutide 2.4 mg versus placebo, in addition to a combined lifestyle intervention (CLI). Both participants and investigators will remain blinded to treatment allocation. A study flowchart is presented in Fig. 1.

### Study population

Patients will be enrolled from 21 hospitals in the Netherlands. Inclusion criteria are symptomatic persistent AF (lasting more than 7 days), first detected within the past six months, with a scheduled electrical cardioversion (ECV), a body mass index (BMI) ≥ 30 kg/m^2^ or BMI ≥ 27 kg/m^2^ with at least one weight-related comorbidity (hypertension, dyslipidaemia, obstructive sleep apnoea, or previously diagnosed cardiovascular disease), age ≥ 18 years, and provision of written informed consent. Key exclusion criteria include paroxysmal, permanent or secondary AF (i.e., AF due to a reversible or transient cause, e.g., post-CABG), prior or current treatment with amiodarone, a planned pulmonary vein isolation (PVI) procedure, prior bariatric surgery, type 1 or type 2 diabetes mellitus, and severe kidney disease, significant valvular heart disease, or HF (NYHA class III or IV). The full inclusion and exclusion criteria are shown in Supplemental Table S1.

### Intervention and control

Participants will receive either semaglutide 2.4 mg or placebo, administered subcutaneously once weekly, for 52 weeks. A titration schedule for semaglutide or placebo will be implemented as follows: 0.24 mg from weeks 1 to 4, 0.5 mg from weeks 5 to 8, 1.0 mg from weeks 9 to 12, 1.7 mg from weeks 13 to 16, and 2.4 mg from weeks 17 to 52. Both groups will participate in a CLI program, a two-year, government-endorsed program consisting of group and individual sessions aimed at weight reduction through dietary modification and increased physical activity. The CLI includes a 500-kcal/day energy deficit and at least 150 min of moderate-intensity physical activity per week or 10,000 steps per day. Dietary and exercise habits will be documented and monitored using diaries or mobile applications.

### Data collection and follow-up

Data are collected via electronic Case Report Forms in Castor (eCRFs), and randomisation is managed through ALEA (IWRS). The primary follow-up period is one year, during which participants attend two in-person study visits: at baseline (randomisation) and at one-year follow-up. At these visits, questionnaires are completed, vital signs are measured, and, where available, laboratory and echocardiographic results are recorded. An ECG is performed at the one-year follow-up visit as part of the primary endpoint assessment.

Five telephone contacts are scheduled during the dose-escalation phase (months 1–4), followed by one additional contact during the maintenance phase.

### Rationale for the co-primary outcome measures

Conventional AF trials usually report binary rhythm outcomes (sinus rhythm versus recurrent AF). However, this approach omits clinical context; two patients in sinus rhythm may have achieved this status through vastly different, costly, or invasive treatment pathways. Therefore, DUTCH-WAIST utilises a hierarchical primary endpoint that integrates rhythm status with therapy escalation. Patients are ranked across seven predefined categories reflecting increasing treatment intensity and clinical severity, while allowing standard care adjustments. The seven outcome levels are, in hierarchical order:

1. Arrhythmic death receiving Vaughan Williams class I or III anti-arrhythmic drugs (most severe).
2. Persistent atrial fibrillation (lack of sinus rhythm) after pulmonary vein isolation (PVI).
3. Persistent atrial fibrillation (lack of sinus rhythm) receiving Vaughan Williams class I or III anti-arrhythmic drugs.
4. Persistent atrial fibrillation (lack of sinus rhythm) without Vaughan Williams class I or III anti-arrhythmic drugs.
5. Sinus rhythm after pulmonary vein isolation (PVI).
6. Sinus rhythm receiving Vaughan Williams class I or III anti-arrhythmic drugs.
7. Sinus rhythm without Vaughan Williams class I or III anti-arrhythmic drugs (least severe).

Given that all included patients have persistent AF requiring cardioversion at baseline, AF documented on the one-year ECG is interpreted as persistent AF.

In addition, a second primary composite endpoint is included to capture the major rhythm-related events that are of clear clinical relevance and consists of persistent atrial fibrillation on ECG at 12 months, catheter ablation for AF, arrhythmic death, or any serious adverse event due to anti-arrhythmic drugs during follow-up.

### Secondary outcome measures

Secondary endpoints include changes in AF-related symptoms, health-related quality of life (measured by the EQ-5D), number of hospitalisations for AF recurrence, unscheduled visits for anti-arrhythmic drugs (AADs) adverse events, cardioversions, exercise behaviour (Behaviour Regulation in Exercise Questionnaire‑2, BREQ-2) and metabolic markers (e.g., weight, BMI, blood pressure, lipid levels, the systemic inflammation marker CRP, and cardiac biomarker NT-proBNP). Economic outcomes, such as the associated total healthcare costs (Medical Consumption Questionnaire, iMCQ), productivity impairment (Work, Productivity and Activity Impairment General Health, WPAI-GH), and quality-adjusted life years (QALYs), will also be assessed.

### Statistical analysis and sample size calculation

The sample size calculation is formally based on the second (binary composite) primary endpoint. This conservative approach was chosen because reliable assumptions regarding patient distribution across the seven hierarchical categories were unavailable at the study design stage. Since ranked endpoints retain more statistical information and are inherently more sensitive than binary endpoints, powering the study based on the binary outcome guarantees adequate statistical power for both co-primary endpoints.

The anticipated composite event rate of 60% in the placebo group was derived from published literature on persistent AF recurrence following electrical cardioversion [6, 7, 12], estimated at 47.5% persistent AF on ECG at 52 weeks, 10% pulmonary vein isolation [15], and 2.5% serious adverse events from anti-arrhythmic drugs [8, 9, 11, 25]. The anticipated reduction to 40% in the semaglutide group was based on evidence that sustained weight loss of ≥ 10% markedly reduces AF recurrence and escalation of rhythm-control therapy [16], combined with semaglutide’s established efficacy in achieving this weight loss [20].

To detect this superiority (60% versus 40%), a total of 280 patients is required to achieve 90% power (using a one-sided Farrington-Manning test). With these same 280 patients, the study maintains 80% power for the first (hierarchical) primary endpoint, assuming an underlying shift in mean ranks of 0.6 (from 4.82 for placebo to 5.42 for semaglutide).

The two primary endpoints will be analysed hierarchically: the first primary endpoint is assessed using the Finkelstein-Schoenfeld method, which compares ranked outcomes for semaglutide- and placebo-treated patients on a pairwise basis. The second primary endpoint will be tested for superiority only if the first primary endpoint shows a statistically significant result to preserve type I error rates. The second primary endpoint will be assessed using a one-sided Farrington-Manning test for equality of proportions.

Analyses will follow the modified intention-to-treat principle, including all randomised patients who received at least one dose of the investigational product, regardless of treatment adherence or subsequent interventions. For patients lost to follow-up or who withdraw consent, endpoint status will be determined based on the last available clinical data or adjudicated events.

Secondary endpoints will be analysed descriptively or using appropriate statistical models. Continuous outcomes will be assessed using mixed-effects models or analysis of covariance, and binary outcomes with logistic regression. Subgroup analyses will be performed for predefined patient groups, including sex, age categories, presence of prior paroxysmal AF at baseline, and left atrial volume index (LAVI), using stratified methods (e.g., Mantel-Haenszel) to explore heterogeneity in treatment effects. These analyses are exploratory and will not be adjusted for multiple comparisons.

A pre-specified sensitivity analysis will be performed in which arrhythmia episodes occurring within the first three months following catheter ablation (the blanking period) are reclassified as sinus rhythm, consistent with standard post-ablation conventions. This analysis will evaluate the robustness of the primary endpoint classification with respect to early post-ablation arrhythmia recurrence.

Safety analyses will be overseen by an independent Data and Safety Monitoring Board (DSMB) throughout the study, which may recommend modifications or early termination of the trial based on safety data. No formal interim efficacy analyses are planned.

### Administrative structure

The study is overseen by a Steering Committee, an independent Data Safety Monitoring Board, and an adjudication committee, with Rijnstate Hospital as sponsor and WCN coordinating study logistics.

### Data management

Data are entered in Castor (eCRF) and randomisation managed via ALEA (IWRS). All data are pseudonymised, stored securely, and archived for at least 15 years.

### Investigational product logistics

A central unblinded pharmacy handles the dispensing and distribution of investigational product via temperature-controlled courier following randomisation through IWRS.

### Patient and public involvement

There was no formal patient or public involvement in the design or conduct of this study. However, the Hartstichting (Dutch Heart Foundation) plays an important role in patient recruitment and serves as a bridge between researchers and the patient community.

### Ethics and dissemination

This study received ethical approval from the Medical Ethics Committee (METC Oost-Nederland). All participants provided written informed consent prior to enrolment. The trial adheres to the Declaration of Helsinki and Good Clinical Practice guidelines to ensure the safety and well-being of participants.

Potential risks include adverse events associated with semaglutide, such as gastrointestinal discomfort and rare but serious conditions like pancreatitis. These risks will be mitigated through regular monitoring and prompt management of side effects. Participants may also benefit from weight loss and improved cardiovascular outcomes, which are anticipated with semaglutide treatment.

## Discussion

### Anticipated results

Based on prior evidence linking weight loss to improved rhythm outcomes, we anticipate that semaglutide as an adjunct to CLI will increase the proportion of patients in sinus rhythm at one year, with fewer AF-related hospitalisations, reduced need for catheter ablation, and lower burden of anti-arrhythmic drug-related adverse events.

### Guidelines-directed atrial fibrillation management

This study aligns with the evolving paradigm of AF management. In the 2024 European Society of Cardiology (ESC) guidelines, [24] management of comorbidities and risk factors now constitute the first pillar of comprehensive AF care, alongside traditional strategies such as avoiding stroke and thromboembolism and reducing symptoms by rate and rhythm control. Furthermore, the guidelines introduce, for the first time, a specific weight-loss target of at least 10%. This shift reflects growing recognition that addressing underlying drivers of AF, such as obesity, is essential, not only because obesity is an independent risk factor for AF, but also because it contributes to the development of hypertension and obstructive sleep apnoea, both of which further increase AF risk and burden.

## Strengths and limitations

Strengths include the randomised, double-blind design minimising bias, a hierarchical primary endpoint providing a nuanced efficacy evaluation, and a sample size calculation ensuring adequate statistical power.

A common challenge with trials involving GLP‑1 receptor agonists is maintaining the double-blind nature. Significant weight loss and associated gastrointestinal side effects in the active treatment group could inadvertently reveal the treatment assignment to both participants and investigators, potentially introducing bias. Additionally, as no protocol-level restrictions on ablation referral were imposed, clinicians aware of a patient’s weight trajectory may have a lower threshold to refer for catheter ablation, potentially introducing differential treatment intensity between groups.

In addition, inclusion and exclusion criteria might contribute to selection bias, potentially limiting the generalisability of the findings to a broader population. For example, participants willing to enrol may represent a subgroup more motivated to pursue weight-loss interventions compared to the general population. The relatively short duration of the trial could also limit the assessment of long-term outcomes and safety profiles.

## Conclusion

This study will evaluate the effect of clinically meaningful weight loss and the potential specific role(s) of semaglutide on the management of AF in patients with overweight or obesity. Ultimately, this trial may support a shift towards more holistic, multi-faceted approaches to treating obesity-related AF, addressing both the arrhythmia and its underlying metabolic drivers.

## Supplementary Information

ESM1: Supplementary material 1

## Data Availability

Not applicable.

## References

[1] Staerk L, Wang B, Preis SR, et al. Lifetime risk of atrial fibrillation according to optimal, borderline, or elevated levels of risk factors: cohort study based on longitudinal data from the Framingham Heart Study. BMJ. 2018;361:k1453. 10.1136/bmj.k1453 PMC5917175 29699974

[2] Marques A, Peralta M, Naia A, et al. Prevalence of adult overweight and obesity in 20 European countries, 2014. Eur J Public Health. 2018;28:295–300. 10.1093/eurpub/ckx143 29036436

[3] Wang TJ, Parise H, Levy D, et al. Obesity and the risk of new-onset atrial fibrillation. JAMA. 2004;292:2471–7. 10.1001/jama.292.20.2471 15562125

[4] Hindricks G, Potpara T, Dagres N, et al. 2020 ESC Guidelines for the diagnosis and management of atrial fibrillation developed in collaboration with the European Association for Cardio-Thoracic Surgery (EACTS). Eur Heart J. 2021;42:373–498. 10.1093/eurheartj/ehaa612 32860505

[5] Seelig J, Chu KG, Derks L, et al. Atriumfibrilleren - diagnoseregistratie. Hart Vaatcijfers. 2020. 118-123.

[6] Frick M, Frykman V, Jensen-Urstad M, Ostergren J, Rosenqvist M. Factors predicting success rate and recurrence of atrial fibrillation after first electrical cardioversion in patients with persistent atrial fibrillation. Clin Cardiol. 2001;24:238–44. 10.1002/clc.4960240313 PMC6655078 11288971

[7] Hemels ME, Van Noord T, Crijns HJ, et al. Verapamil versus digoxin and acute versus routine serial cardioversion for the improvement of rhythm control for persistent atrial fibrillation. J Am Coll Cardiol. 2006;48:1001–9. 10.1016/j.jacc.2006.05.043 16949494

[8] Hohnloser SH, Singh BN. Proarrhythmia with class III antiarrhythmic drugs: definition, electrophysiologic mechanisms, incidence, predisposing factors, and clinical implications. J Cardiovasc Electrophysiol. 1995;6:920–36. 10.1111/j.1540-8167.1995.tb00368.x 8548113

[9] Valembois L, Audureau E, Takeda A, et al. Antiarrhythmics for maintaining sinus rhythm after cardioversion of atrial fibrillation. Cochrane Database Syst Rev. 2019;9:CD005049. 10.1002/14651858.CD005049.pub5 PMC6738133 31483500

[10] Kochiadakis GE, Igoumenidis NE, Marketou ME, et al. Low-dose amiodarone versus sotalol for suppression of recurrent symptomatic atrial fibrillation. Am J Cardiol. 1998;81:995–8. 10.1016/s0002-9149(98)00078-2 9576159

[11] Kirchhof P, Andresen D, Bosch R, et al. Short-term versus long-term antiarrhythmic drug treatment after cardioversion of atrial fibrillation (Flec-SL): a prospective, randomised, open-label, blinded endpoint assessment trial. Lancet. 2012;380:238–46. 10.1016/S0140-6736(12)60570-4 22713626

[12] Pisters R, Nieuwlaat R, Prins MH, et al. Clinical correlates of immediate success and outcome at 1‑year follow-up of real-world cardioversion of atrial fibrillation: the Euro Heart Survey. Europace. 2012;14:666–74. 10.1093/europace/eur406 22223715

[13] Lavie CJ, Pandey A, Lau DH, Alpert MA, Sanders P. Obesity and atrial fibrillation prevalence, pathogenesis, and prognosis: effects of weight loss and exercise. J Am Coll Cardiol. 2017;70:2022–35. 10.1016/j.jacc.2017.09.002 29025560

[14] Mahajan R, Lau DH, Brooks AG, et al. Electrophysiological, electroanatomical, and structural remodeling of the atria as consequences of sustained obesity. J Am Coll Cardiol. 2015;66:1–11. 10.1016/j.jacc.2015.04.058 26139051

[15] Abed HS, Wittert GA, Leong DP, et al. Effect of weight reduction and cardiometabolic risk factor management on symptom burden and severity in patients with atrial fibrillation: a randomized clinical trial. JAMA. 2013;310:2050–60. 10.1001/jama.2013.280521 24240932

[16] Pathak RK, Middeldorp ME, Meredith M, et al. Long-term effect of goal-directed weight management in an atrial fibrillation cohort: a long-term follow-up study (LEGACY). J Am Coll Cardiol. 2015;65:2159–69. 10.1016/j.jacc.2015.03.002 25792361

[17] Wong CX, Ganesan AN, Selvanayagam JB. Epicardial fat and atrial fibrillation: current evidence, potential mechanisms, clinical implications, and future directions. Eur Heart J. 2017;38:1294–302. 10.1093/eurheartj/ehw045 26935271

[18] Look ARG, Wing RR, Bolin P, et al. Cardiovascular effects of intensive lifestyle intervention in type 2 diabetes. N Engl J Med. 2013;369:145–54. 10.1056/NEJMoa1212914 PMC3791615 23796131

[19] LeBlanc ES, Patnode CD, Webber EM, et al. Behavioral and pharmacotherapy weight loss interventions to prevent obesity-related morbidity and mortality in adults: updated evidence report and systematic review for the US Preventive Services Task Force. JAMA. 2018;320:1172–91. 10.1001/jama.2018.7777 PMC13151892 30326501

[20] Wilding JPH, Batterham RL, Calanna S, et al. Once-weekly semaglutide in adults with overweight or obesity. N Engl J Med. 2021;384:989–1002. 10.1056/NEJMoa2032183 33567185

[21] Cataldi M, Cignarelli A, Giallauria F, et al. Cardiovascular effects of antiobesity drugs: are the new medicines all the same? Int J Obes Suppl. 2020;10:14–26. 10.1038/s41367-020-0015-3 PMC7371629 32714509

[22] Helmstädter J, Frenis K, Filippou K, et al. Endothelial GLP‑1 receptor mediates cardiovascular protection by liraglutide in mice with experimental arterial hypertension. Arterioscler Thromb Vasc Biol. 2020;40:145–58. 10.1161/atv.0000615456.97862.30 PMC6946108 31747801

[23] Zhang L, Tian J, Diao S, et al. GLP‑1 receptor agonist liraglutide protects cardiomyocytes from IL-1β-induced metabolic disturbance and mitochondrial dysfunction. Chem Biol Interact. 2020;332:109252. 10.1016/j.cbi.2020.109252 32898504

[24] Van Gelder IC, Rienstra M, Bunting KV, et al. 2024 ESC Guidelines for the management of atrial fibrillation developed in collaboration with the European Association for Cardio-Thoracic Surgery (EACTS). Eur Heart J. 2024;45:3314–414. 10.1093/eurheartj/ehae176 39210723

[25] Van Gelder IC, Hagens VE, Bosker HA, et al. A comparison of rate control and rhythm control in patients with recurrent persistent atrial fibrillation. N Engl J Med. 2002;347:1834–40. 10.1056/NEJMoa021375 12466507

