# Supplementary material for: DUTCH weight control in atrial fibrillation study (DUTCH-WAIST): Design and rationale of a randomised controlled trial
Source: Neth Heart J. 2026 Sep 14;34(10):363–8. doi: 10.1007/s12471-026-02074-2 (PMC13615193; doi:10.1007/s12471-026-02074-2)
Supplement: Supplementary file 1 [file 12471_2026_2074_MOESM1_ESM.docx]

Supplement table 1

**Inclusion criteria**

- Symptomatic, first detected (at maximum 6 months prior to enrollment) persistent AF

- Age ≥ 18

- Obesity, as defined as:

- BMI ≥ 30 kg/m^2^, or
- BMI ≥ 27 kg/m2 with the presence of at least one weight related comorbidity (treated or untreated, e.g. hypertension, dyslipidaemia, obstructive sleep apnea, cardiovascular disease)

- Scheduled ECV

- Written informed consent

**Exclusion criteria**

- Permanent AF

- Secondary AF (due to thyrotoxicosis, infection (e.g. pneumonia) or post-surgery) Current or previous treatment with amiodaron

- Planned pulmonary vein ablation at the time of randomization

- HbA1c ≥ 48 mmol/L, <3 months prior to randomization

- eGFR <30 mL/min/1.73m2

- History of diabetes mellitus type 1 or 2

- Prior bariatric surgery

- Use of other anti-obesity medication, <3 months prior to enrollment

- Contra-indication for, or prior use of a GLP1-receptor agonist*

- History of chronic pancreatitis or acute pancreatitis <6 months

- Acute coronary syndrome <6 months

- Severe (grade III) valvular disease

- Heart failure NYHA class III-IV

- Participation in another investigational drug or device study in the past 30 days (registry enrollment is allowed)

- Any condition or therapy, which would make the participant unsuitable for the study (e.g. vulnerable, non-compliance) or life-expectancy <12 months, as judged by the treating physician.

- Female who is pregnant, breastfeeding, intends to become pregnant, or is of child-bearing potential and not using a highly effective contraceptive method.

* Current absolute contra-indications are hypersensitivity to the active substance or any of the excipients listed in section 6.1 of the SmPC

- Heart failure NYHA class III-IV
- Participation in another investigational drug or device study in the past 30 days (registry enrollment is allowed)
- Any condition or therapy, which would make the participant unsuitable for the study (e.g. vulnerable, non-compliance) or life-expectancy <12 months, as judged by the treating physician.
- Female who is pregnant, breastfeeding, intends to become pregnant, or is of child-bearing potential and not using a highly effective contraceptive method.

* Current absolute contra-indications are hypersensitivity to the active substance or any of the excipients listed in section 6.1 of the SmPC
